# Cost-effectiveness of TAS-102 plus bevacizumab versus TAS-102 monotherapy in patients with metastatic colorectal cancer

**DOI:** 10.1186/s12876-021-01771-z

**Published:** 2021-04-20

**Authors:** Kiyoaki Sugiura, Yuki Seo, Takayuki Takahashi, Hideyuki Tokura, Yasuhiro Ito, Motomu Tanaka, Norihiro Kishida, Yusuke Nishi, Yoshihiko Onishi, Hikaru Aoki

**Affiliations:** Ashikaga Red Cross Hospital, 284-1 Yobe-cho, Ashikaga-shi, Tochigi 326-0843 Japan

**Keywords:** Colorectal cancer, TAS-102, Bevacizumab, Cost-effectiveness

## Abstract

**Background:**

TAS-102 plus bevacizumab is an anticipated combination regimen for patients who have metastatic colorectal cancer. However, evidence supporting its use for this indication is limited. We compared the cost-effectiveness of TAS-102 plus bevacizumab combination therapy with TAS-102 monotherapy for patients with chemorefractory metastatic colorectal cancer.

**Method:**

Markov decision modeling using treatment costs, disease-free survival, and overall survival was performed to examine the cost-effectiveness of TAS-102 plus bevacizumab combination therapy and TAS-102 monotherapy. The Japanese health care payer’s perspective was adopted. The outcomes were modeled on the basis of published literature. The incremental cost-effectiveness ratio (ICER) between the two treatment regimens was the primary outcome. Sensitivity analysis was performed and the effect of uncertainty on the model parameters were investigated.

**Results:**

TAS-102 plus bevacizumab had an ICER of $21,534 per quality-adjusted life-year (QALY) gained compared with TAS-102 monotherapy. Sensitivity analysis demonstrated that TAS-102 monotherapy was more cost-effective than TAS-102 and bevacizumab combination therapy at a willingness-to-pay of under $50,000 per QALY gained.

**Conclusions:**

TAS-102 and bevacizumab combination therapy is a cost-effective option for patients who have metastatic colorectal cancer in the Japanese health care system.

## Introduction

In Japan, approximately 150,000 new patients per year are currently diagnosed as colorectal cancer (CRC), and it was the second largest cause of cancer-related death in 2018 [1]. Despite advancements in the treatment of metastatic CRC (mCRC), survival rates remain poor, and the expected survival without effective pharmacologic treatment is approximately 6 months [2–4]. TAS-102 (trifluridine/tipiracil) is an orally anti-cancer drug containing a thymidine analog (trifluridine). TAS-102 significantly improves overall survival and progression-free survival of patients with mCRC who is refractory to standard therapies [2, 3, 5]. Recently, potential combination regimens containing TAS-102 have attracted attention. The anti-VEGF antibody bevacizumab is expected to be an excellent anti-cancer agent when added to TAS-102 in patients who have chemorefractory mCRC. The C-TASK FORCE conducted a phase 1/2 trial that provided evidence endorsing the clinical use of TAS-102 plus bevacizumab in patients with unresectable mCRC [6]. Additionally, a phase 2 trial was conducted by Pfeiffer et al. which detailing the promising activity of TAS-102 plus bevacizumab compared to TAS-102 monotherapy in patients with mCRC [7]. Despite the absence of a phase 3 trial, these data provided a rationale for the clinical use of TAS-102 plus bevacizumab in patients who have chemorefractory mCRC. However, to our knowledge, there is no cost-effectiveness analyses evaluating TAS-102 plus bevacizumab combination therapy. Thus, the present study compared the cost-effectiveness of TAS-102 plus bevacizumab combination therapy with TAS-102 monotherapy for patients with chemorefractory mCRC.

## Materials and methods

**Fig. 1:**
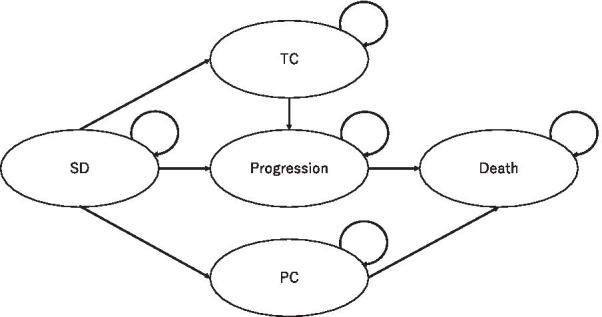
Markov model diagrams for patients undergoing monotherapy and combination therapy. Five health states exist: stable disease (SD), treatment complication (TC), progression, progression with complication (PC) and death. Each circles represents different health states. Patients in the model move between the health states following the direction of the arrow and assigned transition-probabilities for every model-cycle. Circular arrows indicate transition-probability for remaining in the same state for the next model-cycle. Death is the absorbing state

A Markov decision model simulating costs and quality-adjusted life-years (QALYs) related to TAS-102 plus bevacizumab combination therapy and TAS-102 monotherapy was constructed using R, version 3.4.3 with the heemod package (R Foundation for Statistical Computing, Vienna, Austria). The reference case was an adult meeting the C-TASK FORCE inclusion criteria [6]. The model assumes that patients move through five possible states: stable disease (SD), treatment with complications, progression, progression with complications, and death (Fig. 1). Patients start in the SD state in the first Markov cycle and move to other states on the basis of the set transition probabilities calculated from published data (Table 1) [6, 7]. The Declining Exponential Approximation of Life Expectancy method was used to convert median overall survival and progression-free survival to rates and their respective transition probabilities [8]. Eight weeks, which was adopted as the interval between status assessments in the trials was used as Markov cycle length [6, 7]. The model ran for 30 cycles, corresponding to 60 months of follow-up. Annual rate of 3.5% was discounted from costs and QALYs, in line with NICE guidance [9]. For simplicity, all patients began in the SD state, and they were moved to the death state only through a progression state. Only grade 3–4 complications with incidence rates exceeding 5% were considered in this model. The costs of managing complications were counted once in the clinical scenario of each patient in the model. No patients discontinued treatment because of chemotherapy-related complications because the trials did not mention dropouts caused by complications [6, 7]. The study was modelled using only data from publically available PubMed database. Therefore, there was no requirement of institutional board approval or patient consent. This study is reported based on the CHEERS reporting guidelines [10].

**Table 1:** Model variables: transition probabilities

	Sensitivity analysis			
	Base value	Minimum	Maximum	Distribution
Combination therapy				
Progression	0.38191794	0.28643845	0.47739742	Binomial
Death	0.18161864	0.13621398	0.2270233	Binomial
Complication	0.0846049	0.06345368	0.10575613	Binomial
Monotherapy				
Progression	0.55459395	0.41594546	0.69324243	Binomial
Death	0.26230083	0.19672562	0.32787604	Binomial
Complication	0.1126958	0.08452185	0.14086975	Binomial

### Cost

In this study, only direct medical costs were included based on the health care payer’s perspective. The social insurance reimbursement schedule and drug tariff of the fee-for-service system in Japan was used for cost calculation [11, 12]. All costs were expressed based on US dollars (1 US dollar = 110.05 ¥, 2019). The drug costs of bevacizumab and TAS-102 were derived on the basis of the assumed body weight (65 kg) and body surface area (1.72 m^2^) of the patients. Frequency-weighted complication costs were derived on the basis of the trials [6, 7]. Costs were determined on the basis of the treatment drugs (e.g., per-unit costs: filgrastim for neutropenia, $68.29; cefepime for febrile neutropenia, $5.96). No costs were assigned to all progression and death states. It is assumed that costs for care after progression and for other routine care (best supportive care, office visits, imaging) were similar between the two arms. Hence, we did not include them in this analysis. The costs used in the model are presented in Table 2.

**Table 2:** Model variables: direct medical costs

	Sensitivity analysis			
	Base value ($)	Minimum ($)	Maximum ($)	Distribution ($)
TAS-102	1833.66516	1375.24887	2292.08145	Gamma
Bevacizumab	767.945701	575.959276	959.932127	Gamma
Outpatient chemotherapy				
IV drip fee	65.6108597	49.2081448	82.0135747	Gamma
Administration fee for chemotherapy	36.199095	27.1493213	45.2488688	Gamma
Outpatient service fee	6.60633484	4.95475113	8.25791855	Gamma
Prescription fee	3.80090498	2.85067873	4.75113122	Gamma
Administration fee for chemotherapy(monotherapy)	10.4072398	7.80542986	13.0090498	Gamma
Administration fee for chemotherapy (combination)	220.633484	165.475113	275.791855	Gamma
Costs for management of complication (monotherapy)	28.7519014	21.5639261	35.9398768	Gamma
Costs for management of complication (combination)	72.0921038	54.0690778	90.1151297	Gamma

### Utility

None of the TAS-102 studies directly considered health state utility. Therefore, the utility of patients receiving TAS-102 monotherapy was assumed to match that reported in the CORRECT study of regorafenib, in which the health state utility of patients with chemorefractory mCRC treated with an oral anti-cancer agent was measured [13]. Similarly, because of the lack of evidence for the health state utility values of patients treated with TAS-102 and bevacizumab, the assumption was adopted in the combination arm based on other trials in which oral and infusion anti-cancer agents were used for patients with chemorefractory mCRC [14–16]. The utilities in each state are presented in Table 3.

**Table 3:** Model variables: utilities

	Sensitivity analysis			
	Base value	Minimum	Maximum	Distribution
Combination therapy				
Stable disease	0.72	0.54	0.9	Binomial
Treatment complication	0.72	0.54	0.9	Binomial
Progression	0.59	0.4425	0.7375	Binomial
Progression with complication	0.59	0.4425	0.7375	Binomial
Death	0	0	0	Binomial
Monotherapy				
Stable disease	0.73	0.5475	0.9125	Binomial
Treatment complication	0.73	0.5475	0.9125	Binomial
Progression	0.59	0.4425	0.7375	Binomial
Progression with complication	0.59	0.4425	0.7375	Binomial
Death	0	0	0	Binomial

### Cost-effective analysis

The primary outcome was the incremental cost-effectiveness ratio (ICER) between the two treatment regimens. The threshold in the analysis was set at $117,746/QALY gained (threefold per capita GDP of Japan, 2018) in compliance with the World Health Organization (WHO) guideline for cost-effectiveness [17, 18].

### Sensitivity analysis

Deterministic and probabilistic sensitivity analyses were performed to evaluate the uncertainty of the model. In deterministic sensitivity analyses, all parameters varied by 25%. In probabilistic sensitivity analyses, all model parameters varied simultaneously with probability distributions with 1000 re-samplings. Each variable’s range and distribution are listed in Tables 1, 2 and 3.

## Results

### Base case results

The cost and utilities per 8-week Markov cycle in each state are presented in Table 4. Compared with TAS-102 monotherapy, the base case results in the Table 5 showed that TAS-102 plus bevacizumab combination therapy had an ICER of $21,534 per QALY gained. At the Japanese willingness-to-pay (WTP) threshold of $117,912 per QALY, The base case results suggested that TAS-102 plus bevacizumab was cost-effective compared to TAS-102 monotherapy.

**Table 4:** Costs and utilities per 9-week Markov cycle for each model state

State	Combination arm		Monotherapy arm	
	Cost/8 week ($)	QALY/8 week	Cost/8 week ($)	QALY/8 week
Stable disease	793,432.000	0.11076923	407,540.000	0.11230769
Treatment complication	801,398.177	0.11076923	410,717.085	0.11230769
Progression	00	0.09076923	0	0.09076923
Progression with complication	7966.17746	0.09076923	3177.08511	0.09076923
Death	0	0	0	0

**Table 5:** Results of base case analysis

	Total direct medical cost ($)	QALY	Incremental cost ($)	Incremental QALY	ICER($/QALY)	Ref
Monotherapy	3434.834	0.501	Ref	Ref	Ref	–
Combination therapy	8424.188	0.732	4,989.354	0.231	21,553.630	Monotherapy

### Sensitivity analysis

**Fig. 2:**
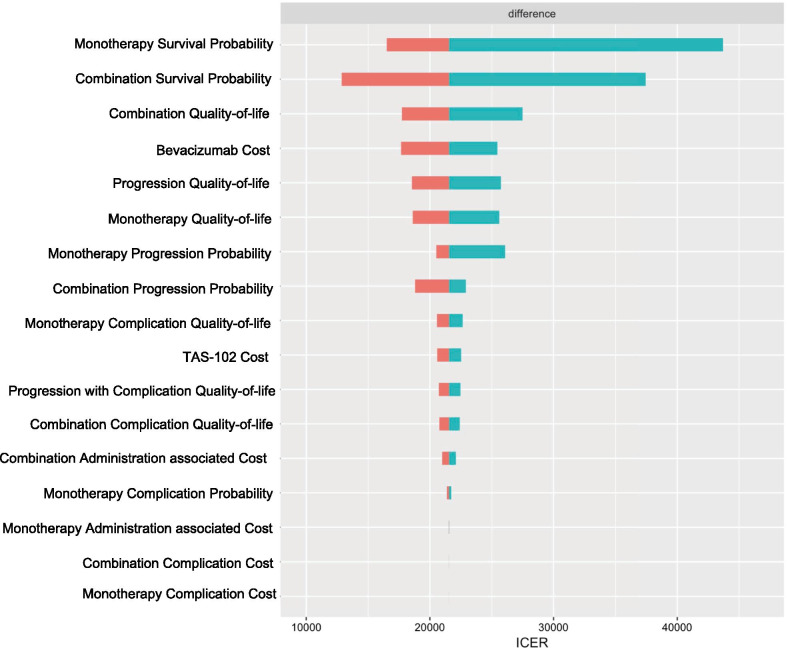
Tornado diagram for deterministic sensitivity analysis. Plot shows the range in incremental cost-effectiveness ratio (ICER) when each variables is tested between at the maximum and the minimum values. Variables to which ICER were not sensitive are not shown

**Fig. 3:**
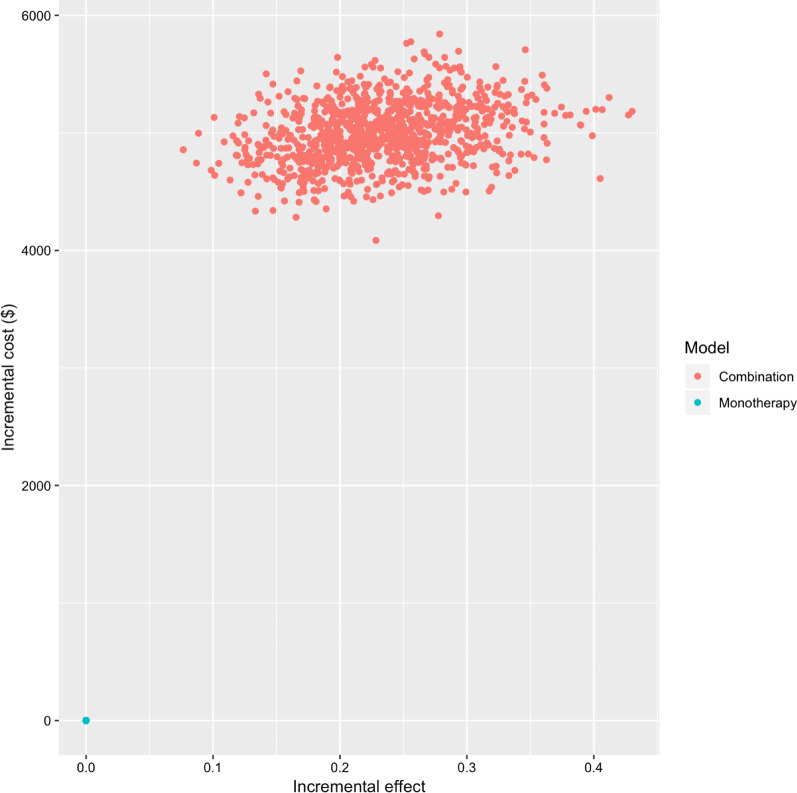
Probabilistic sensitivity analysis. Plot of incremental quality-adjusted life-years (QALYs) versus incremental cost from 1000 re-samplings varying all model parameters with probability distributions

**Fig. 4:**
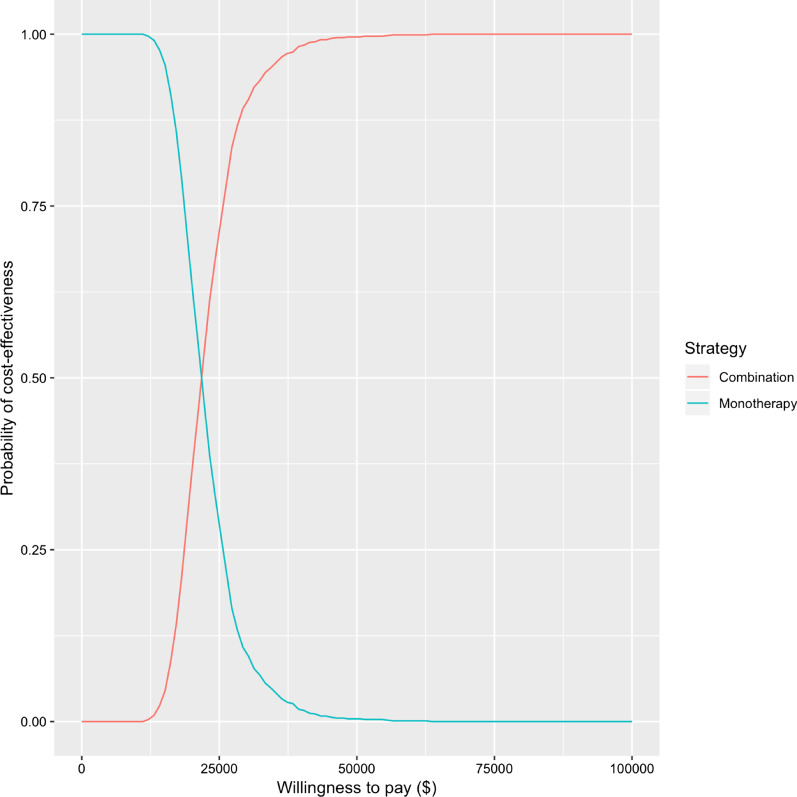
Cost-effectiveness acceptability curve for probabilistic sensitivity analyses. Plot of the probability of cost-effectiveness for TAS-102 monotherapy and TAS-102 plus bevacizumab combination therapy versus different willingness-to-pay values

The tornado diagram of deterministic sensitivity analysis in Fig. 2 reveals the parameters most strongly influencing the ICER difference between TAS-102 monotherapy and TAS-102 and bevacizumab combination therapy. ICER was predominantly influenced by the transition probability for survival, patient’s utility in the SD state during combination therapy, and the cost of bevacizumab. The cost of management for complication had ignorable impact on the ICER. The result of probabilistic sensitivity analysis showed that all re-samplings had a positive ICER, indicating better QALY with high cost in the combination therapy group. Figure 3 presented the plot. Using the cost-effectiveness acceptability curve in Fig. 4, it is demonstrated that TAS-102 monotherapy was more cost-effective than TAS-102 and bevacizumab combination therapy at a WTP of less than $50,000 per QALY. However, the combination therapy was considered cost-effective at higher WTP thresholds.

## Discussion

The results of this study suggested that TAS-102 plus bevacizumab combination therapy had an ICER of $21,534 per QALY gained, making this regimen more cost-effective than TAS-102 monotherapy for patients with mCRC at the threshold set from the Japanese health care payer’s perspective. In Japan, it is not routinely recommended to administer bevacizumab together with third-line TAS-102 monotherapy in patients with mCRC. This study revealed that TAS-102 plus bevacizumab combination therapy is a properly priced treatment with high cost-effectiveness in the health care system in Japan.

Previously, the efficacy of adding bevacizumab to the treatment regimen for patients with CRC was investigated in other cost-effectiveness studies. Goldstein et al. reported that adding bevacizumab to FOLFOX in the first-line therapy provided an ICER of $571,240 per QALY gained, and adding bevacizumab to FOLFIRI in the second-line therapy provided an ICER of $364,083 per QALY gained [19]. Zhang et al. found that the ICER gained by capecitabine plus bevacizumab combination therapy compared to capecitabine monotherapy in elderly patients with mCRC was $95,564.33 per QALY [20]. Tappenden et al. stated that the ICER gained by adding bevacizumab to IFL (irinotecan plus FU/LV) is approximately £62,857 per QALY, and by adding bevacizumab to 5-FU/LV is approximately £88,436 per QALY [21]. These studies concluded that the adding bevacizumab to the chemotherapeutic regimen is unlikely to be cost-effective. Unlike these studies, our results suggested that TAS-102 plus bevacizumab combination therapy was cost-effective compared with TAS-102 alone. One possible reason for our finding is that bevacizumab costs less in Japan than in other countries. In the aforementioned studies, the prices of bevacizumab were $6.462/mg, $2.95/mg, and £2.31/mg [19–21]. Contrarily, bevacizumab costs $1.92/mg in Japan. Even considering the differences of the base case body size and treatment regimen between studies, the difference in cost for adding bevacizumab may be a factor making TAS-102 plus bevacizumab combination therapy cost-effective in the Japanese health care payer’s perspective. Our results suggested that if the cost of bevacizumab can be set to the its Japanese cost in other countries, the addition of bevacizumab would be cost-effective in these countries. It is an important health care problem to set the price of expensive drugs on the basis of cost-effectiveness. Our findings suggested that governments may need to adjust the price of bevacizumab for patients with mCRC.

The phase 1/2 and phase 2 trials of TAS-102 and bevacizumab for patients mCRC reported that median overall survival might be increased by approximately 2.4 and 2.7 months, respectively, in patients receiving combination therapy [6, 7]. The results for small and varied populations might not be generalizable to the Japanese health care system. However, this increase did not differ from the previous results of clinical trials describing the clinical benefits of adding bevacizumab to monotherapy for patients with mCRC [14, 22]. As supported by the results of sensitivity analysis, if we adjusted the transition probability on the basis of future large randomized studies, it is quite possible that the ICER would become similar to the base case results. Until large clinical trials directly investigating this combination for patients with mCRC are conducted, this study supports the clinical benefit of the TAS-102 plus bevacizumab combination therapy for patients with mCRC.

In the UK, NICE guideline recommends that the ICER threshold should be £20,000–30,000 per QALY gained [9]. In the United States, a QALY threshold of $50,000–100,000 is often used. However, to date, no established cost-effectiveness threshold is available in Japan. Therefore, the WHO’s WTP recommendation was used for ICER thresholds in our model. This threshold is a numerical value to be used solely as a common cognitive anchor rather than as an indicator for restraining clinical decision-making. However, we consider the results of the sensitivity analyses supports the robustness of our conclusion in this study. The acceptability curve revealed that at the WTP was $20,000, the probability of TAS-102 and bevacizumab combination therapy being the most cost-effective strategy was approximately 50%. The threshold is adequately lower than ICER according to WHO’s recommendation and similar to the ICER thresholds in other countries. Hence, the results in this study demonstrated that the combination regimen was cost-effective in the range of possible WTP values for the Japanese health care payers.

Deterministic sensitivity analyses suggested that change in the transition probability of progression to death in both arms had a significant influence on ICER. However, the variation of ICER falls in the range of $10,000–50,000, falling below the ICER threshold set in this study, even if the transition probability is varied by ± 25%. Additionally, probabilistic sensitivity analysis demonstrated that the WTP was still within the ICER threshold range. Taken together, the sensitivity analysis suggested that the results of this study would be robust in real-world clinical practice. However, future clinical phase 3 trials evaluating the oncological effects of the TAS-102 and bevacizumab combination are needed.

This study had several limitations. First, we calculated parameters in our model from phase 1/2 trials with small numbers of patients [6, 7]. However, the favorable results of these trials, which encourage the use of TAS-102 plus bevacizumab, are worth considering. There are no effective chemotherapeutics and tumor rarely regress in the third-line setting [23]. Therefore, new treatment regimens for patients with mCRC are urgently needed. Recently, the clinical efficacy and safety of the combination of TAS-102 plus bevacizumab are also reported by several retrospective studies, suggesting that this regimen is potentially promising option for patients with mCRC [24, 25]. Although the benefit of TAS-102 plus bevacizumab has not been proved clearly in a phase 3 clinical trial, this is not a sufficient ground to refrain from using TAS-102 plus bevacizumab for patients with mCRC in clinical practice. Additionally, sensitivity analysis demonstrated that even if the parameters varies, the results are robust. We hope current study help for Japanese health insurance and guidelines discuss the inclusion of the combination therapy. Second, utility was not assessed in the trials. Hence, we assumed health-related utility associated with the disease and complications from indirect sources. Although the robustness of the results are supported by our sensitivity analysis, the accuracy of our analysis still may be questioned by this limitation. Future research should investigate the utility of the combination therapy in patients with mCRC. Third, in this cost-effectiveness analysis, the Japanese societal health care payer’s perspective was adopted, and the results may not be generalizable to other insurers in other countries. However, our model provides evidence that TAS-102 and bevacizumab can be cost-effective in health economic systems with proper pricing.

## Conclusions

This study demonstrated that TAS-102 and bevacizumab combination therapy is a cost-effective treatment option for patients with mCRC in the Japanese health care system. Our study provided evidence for evaluating this regimen for the treatment of mCRC from a Japanese health care payer’s perspective. The results might influence decision-making for patients, the government, and healthcare financial structures. Further information based on clinical trials is needed to evaluate the cost-effectiveness of TAS-102 and bevacizumab in patients with mCRC.

## Data Availability

The datasets generated and/or analysed during this study are available from the corresponding author on reasonable request.

## Abbreviations

CRC: Colorectal cancer
ICER: Incremental cost-effectiveness ratio
mCRC: Metastatic colorectal cancer
QALY: Quality-adjusted life-year
SD: Stable disease
WTP: Willingness-to-pay

## References

[1] Cancer Statistics in Japan. https://ganjoho.jp/reg_stat/statistics/stat/summary.html. 26, September 2020.

[2] Mayer RJ, Van Cutsem E, Falcone A, Yoshino T, Garcia-Carbonero R, Mizunuma N, Yamazaki K, Shimada Y, Tabernero J, Komatsu Y, Sobrero A, Boucher E, Peeters M, Tran B, Lenz HJ, Zaniboni A, Hochster H, Cleary JM, Prenen H, Benedetti F, Mizuguchi H, Makris L, Ito M, Ohtsu A (2015). Randomized trial of TAS-102 for refractory metastatic colorectal cancer. N Engl J Med.

[3] Yoshino T, Mizunuma N, Yamazaki K, Nishina T, Komatsu Y, Baba H, Tsuji A, Yamaguchi K, Muro K, Sugimoto N, Tsuji Y, Moriwaki T, Esaki T, Hamada C, Tanase T, Ohtsu A (2012). TAS-102 monotherapy for pretreated metastatic colorectal cancer: a double-blind, randomised, placebo-controlled phase 2 trial. Lancet Oncol.

[4] Hoyle M, Peters J, Crathorne L, Jones-Hughes T, Cooper C, Napier M, Hyde C (2013). Cost-effectiveness of cetuximab, cetuximab plus irinotecan, and panitumumab for third and further lines of treatment for KRAS wild-type patients with metastatic colorectal cancer. Value Health.

[5] Xu J, Kim TW, Shen L, Sriuranpong V, Pan H, Xu R, Guo W, Han SW, Liu T, Park YS, Shi C, Bai Y, Bi F, Ahn JB, Qin S, Li Q, Wu C, Ma D, Lin D, Li J (2018). Results of a randomized, double-blind, placebo-controlled, phase III trial of trifluridine/tipiracil (TAS-102) monotherapy in Asian patients with previously treated metastatic colorectal cancer: the TERRA study. J Clin Oncol.

[6] Kuboki Y, Nishina T, Shinozaki E, Yamazaki K, Shitara K, Okamoto W, Kajiwara T, Matsumoto T, Tsushima T, Mochizuki N, Nomura S, Doi T, Sato A, Ohtsu A, Yoshino T (2017). TAS-102 plus bevacizumab for patients with metastatic colorectal cancer refractory to standard therapies (C-TASK FORCE): an investigator-initiated, open-label, single-arm, multicentre, phase 1/2 study. Lancet Oncol.

[7] Pfeiffer P, Yilmaz M, Möller S, Zitnjak D, Krogh M, Petersen LN, Poulsen L, Winther SB, Thomsen KG, Qvortrup C (2020). TAS-102 with or without bevacizumab in patients with chemorefractory metastatic colorectal cancer: an investigator-initiated, open-label, randomised, phase 2 trial. Lancet Oncol.

[8] Beck JR, Kassirer JP, Pauker SG (1982). A convenient approximation of life expectancy (the "DEALE"). I. Validation of the method. Am J Med.

[9] Guide to the Methods of Technology Appraisal 2013. https://www.ncbi.nlm.nih.gov/books/NBK395867/pdf/Bookshelf_NBK395867.pdf. September 23 2020. 27905712

[10] Husereau D, Drummond M, Petrou S, Carswell C, Moher D, Greenberg D, Augustovski F, Briggs AH, Mauskopf J, Loder E (2013). Consolidated health economic evaluation reporting standards (CHEERS) statement. BMJ.

[11] Iyaku-Hoho-Kenkyujo.Inc. National Health Insurance Drug Price Standard. Tokyo, Japan: Jiho,Inc: 2016.

[12] Laboratory SIR. Reimbursement Schedule of Social Insurance.Tokyo. Tokyo, Japan: Social Insurance Research Laboratory: 2016.

[13] Grothey A, Van Cutsem E, Sobrero A, Siena S, Falcone A, Ychou M, Humblet Y, Bouché O, Mineur L, Barone C, Adenis A, Tabernero J, Yoshino T, Lenz HJ, Goldberg RM, Sargent DJ, Cihon F, Cupit L, Wagner A, Laurent D (2013). Regorafenib monotherapy for previously treated metastatic colorectal cancer (CORRECT): an international, multicentre, randomised, placebo-controlled, phase 3 trial. Lancet.

[14] Simkens LH, van Tinteren H, May A, ten Tije AJ, Creemers GJ, Loosveld OJ, de Jongh FE, Erdkamp FL, Erjavec Z, van der Torren AM, Tol J, Braun HJ, Nieboer P, van der Hoeven JJ, Haasjes JG, Jansen RL, Wals J, Cats A, Derleyn VA, Honkoop AH, Mol L, Punt CJ, Koopman M (2015). Maintenance treatment with capecitabine and bevacizumab in metastatic colorectal cancer (CAIRO3): a phase 3 randomised controlled trial of the Dutch Colorectal Cancer Group. Lancet.

[15] Sherman SK, Lange JJ, Dahdaleh FS, Rajeev R, Gamblin TC, Polite BN, Turaga KK (2019). Cost-effectiveness of maintenance capecitabine and bevacizumab for metastatic colorectal cancer. JAMA Oncol.

[16] Quidde J, Hegewisch-Becker S, Graeven U, Lerchenmüller CA, Killing B, Depenbusch R, Steffens CC, Lange T, Dietrich G, Stoehlmacher J, Reinacher A, Tannapfel A, Trarbach T, Marschner N, Schmoll HJ, Hinke A, Al-Batran SE, Arnold D (2016). Quality of life assessment in patients with metastatic colorectal cancer receiving maintenance therapy after first-line induction treatment: a preplanned analysis of the phase III AIO KRK 0207 trial. Ann Oncol.

[17] Statistics. https://www.esri.cao.go.jp/jp/sna/data/data_list/kakuhou/files/h30/sankou/pdf/hitoriatarigdp_20191226.pdf. 26, September 2020.

[18] Murray CJ, Evans DB, Acharya A, Baltussen RM (2000). Development of WHO guidelines on generalized cost-effectiveness analysis. Health Econ.

[19] Goldstein DA, Chen Q, Ayer T, Howard DH, Lipscomb J, El-Rayes BF, Flowers CR (2015). First- and second-line bevacizumab in addition to chemotherapy for metastatic colorectal cancer: a United States-based cost-effectiveness analysis. J Clin Oncol.

[20] Zhang PF, Wen F, Zhou J, Huang JX, Zhou KX, Wu QJ, Wang XY, Zhang MX, Liao WT, Li Q (2020). Cost-effectiveness analysis of capecitabine plus bevacizumab versus capecitabine alone in elderly patients with previously untreated metastatic colorectal cancer from Chinese societal perspective. Clin Transl Oncol.

[21] Tappenden P, Jones R, Paisley S, Carroll C (2007). The cost-effectiveness of bevacizumab in the first-line treatment of metastatic colorectal cancer in England and Wales. Eur J Cancer(Oxford, England : 1990).

[22] Cunningham D, Lang I, Marcuello E, Lorusso V, Ocvirk J, Shin DB, Jonker D, Osborne S, Andre N, Waterkamp D, Saunders MP (2013). Bevacizumab plus capecitabine versus capecitabine alone in elderly patients with previously untreated metastatic colorectal cancer (AVEX): an open-label, randomised phase 3 trial. Lancet Oncol.

[23] Nielsen DL, Palshof JA, Larsen FO, Jensen BV, Pfeiffer P (2014). A systematic review of salvage therapy to patients with metastatic colorectal cancer previously treated with fluorouracil, oxaliplatin and irinotecan +/- targeted therapy. Cancer Treat Rev.

[24] Fujii H, Matsuhashi N, Kitahora M, Takahashi T, Hirose C, Iihara H, Yamada Y, Watanabe D, Ishihara T, Suzuki A, Yoshida K (2020). Bevacizumab in combination with TAS-102 improves clinical outcomes in patients with refractory metastatic colorectal cancer: a retrospective study. Oncologist.

[25] Kotani D, Kuboki Y, Horasawa S, Kaneko A, Nakamura Y, Kawazoe A, Bando H, Taniguchi H, Shitara K, Kojima T, Tsuji A, Yoshino T (2019). Retrospective cohort study of trifluridine/tipiracil (TAS-102) plus bevacizumab versus trifluridine/tipiracil monotherapy for metastatic colorectal cancer. BMC Cancer.

